# A ß-Secretase Modulator Decreases Tau Pathology and Preserves Short-Term Memory in a Mouse Model of Neurofibrillary Degeneration

**DOI:** 10.3389/fphar.2021.679335

**Published:** 2021-06-29

**Authors:** Marie Tautou, Sabiha Eddarkaoui, Florian Descamps, Paul-Emmanuel Larchanché, Jamal El Bakali, Liesel Mary Goveas, Mélanie Dumoulin, Chloé Lamarre, David Blum, Luc Buée, Patricia Melnyk, Nicolas Sergeant

**Affiliations:** ^1^ Inserm, CHU Lille, U1172–LilNCog–Lille Neuroscience and Cognition, University of Lille, Lille, France; ^2^ Alzheimer and Tauopathies, LabEx DISTALZ, Lille, France

**Keywords:** Alzheimer’s disease, BACE protein, lysosomes, proteostasis, tauopathy, tau pathology

## Abstract

Identifying which among several *in cellulo* pharmacological activities is necessary for the proper *in vivo* activity is essential for further drug development against Alzheimer’s disease pathophysiological processes. An in-depth structure–activity relationship–based study has been carried out, and two molecules, named MAGS02-14 and PEL24-199, that share a ß-secretase modulatory effect associated or not to a lysosomotropic activity in cellulo have been identified. In terms of chemical formulas, MAGS02-14 and PEL24-199 only differ from each other by a single nitrogen atom. The study aimed to elucidate the *in vivo* pharmacological effects of lysosomotropic and/or the ß-secretase modulatory activity in a tau pathology mouse model. To address this question, the THY-Tau22 transgenic model of tauopathy was treated with both compounds for 6 weeks in a curative paradigm. Short-term memory, tau burden, and inflammatory processes were analyzed using orthogonal methods, and PEL24-199, but not MAGS02-14, was shown to restore the short-term memory and reduce the neurofibrillary degenerating process. These effects were associated with a reduced phosphorylation of tau, an increased phosphatase expression, and decreased astrogliosis. Our results, therefore, suggest that the lysosomotropic activity may be nonessential for the effect on tau pathology.

## Introduction

Alzheimer’s disease (AD) is a neurodegenerative disease defined by the presence of two neuropathological brain lesions: intraneuronal aggregates of tau proteins and extracellular deposition of toxic Aβ peptides, respectively, referred to as tau and amyloid pathologies. Aβ peptides are generated by sequential cleavages of the amyloid precursor protein (APP). The ß-secretase (BACE1) endoprotease catalyzes the first N-terminal cleavage followed by a second γ-secretase endoproteolytic C-terminal cleavage of Aβ peptides (for a review, see Müller et al., 2017). Tau pathology corresponds to the progressive accumulation and aggregation of abnormally and hyperphosphorylated isoforms of the microtubule-associated protein tau, *in fine* forming the so-called neurofibrillary tangles (NFTs) (Buee et al., 2000; Liu et al., 2012; Gao et al., 2018). The neuropathological lesions and cognitive impairments are a primary specific criterion to the definition and diagnosis of AD, suggesting that the pathophysiological processes underlying the development of these lesions are tightly linked to the disease and distinguish AD from other neurodegenerative diseases.

An accumulating body of evidence suggests that APP metabolism regulates tau expression *via* the inhibition of ß-secretase which reduces intracellular tau protein. The cellular protein homeostasis systems that are regulated by autophagy and the endosome/lysosome pathways may lie at the crossroads of APP and tau metabolism. (Bourdenx et al., 2021). These degradation systems play a central role in removing misfolded proteins (Frake et al., 2015). Perturbed trafficking of lysosomal vesicles and enzymes, and the intravesicular accumulation of substrates are characteristics of lysosomal storage disorders. Several other such dysfunctions of the lysosomal system that further implicate a dysfunction of the proteostasis systems (Nixon and Yang, 2011; Piras et al., 2016) have been reported in AD and tauopathies. The autophagic flow leading to autophagosome formation through the fusion of autophagosomes with lysosomes is a key process that can be blocked by lysosomotropic agents such as chloroquine (Tam et al., 2014; Mauthe et al., 2018).

We previously described molecules having a chloroquinoline nucleus substituted with an N, N’-disubstituted piperazine moiety. This family of molecules acts on the autophagic/endolysosomal systems, some of which were shown to be effective against both amyloid and tau pathologies *in vitro* and *in vivo* (Melnyk et al., 2015; Sergeant et al., 2019). A ligand-based approach enabled us to determine a pharmacophore and synthesize multiple compounds with different scaffolds derived from this pharmacophore (Gay et al., 2018). Among these new compounds, two differ by a single nitrogen atom [MAGS02-14 compound 30 in Gay et al. (2018)] substituted by a carbon atom at the same position for PEL24-199 [compound 31 in Gay et al. (2018)]. Although having a different chemical structure, MAGS02-14 exhibits a lysosomotropic activity comparable to chloroquine and a ß-secretase non competitive inhibitory activity *in cellulo*. In contrast, PEL24-199 only has a non competitive ß-secretase inhibitory activity with a strongly reduced lysosomotropic activity. The MAGS02-14–treated cells also exhibit swelling of the intracellular vesicles and accumulation of LC3 and p62 markers indicative of an autophagy flux inhibition. Expression and localization of these markers are not modified by PEL24-199 treatment, while Aβ_1-40_/Aβ_1-42_ production is reduced in both MAGS02-14 and PEL24-199 (Gay et al., 2018). This autophagic flux inhibition of MAGS02-14 and the absence of lysosomotropic activity for PEL24-199 can then be associated with a shared ß-secretase non competitive inhibiting effect *in cellulo*.

Modulation of APP metabolism using either ß- or γ-secretase inhibitors regulates the dosage of tau protein in human-derived cerebral cortical neurons (Moore et al., 2015). Moreover, we previously demonstrated that molecules used for the scaffold design of MAGS02-14 and PEL24-199 showed efficacy to reduce both amyloid and tau pathologies *in vivo* in a preventive paradigm (Sergeant et al., 2019). This study has been carried out to investigate whether MAGS02-14 and/or PEL24-199 could reverse the tau pathology through an *in vivo* study on a mouse model of hippocampal NFTs. Through this study, we aimed to identify which among the lysosomotropic or ß-secretase modulatory activity is pivotal to the improvement of the cognitive function and associated tau pathology.

## Materials and Methods

### Animals

In this study, we used females THY-Tau22 transgenic and wild-type (WT) littermates (C57Bl/6J genetic background), obtained by crossing THY-Tau22 heterozygous males (C57Bl/6J) with WT females. All animals were housed in a pathogen-free facility with a 12/12 h light–dark cycle and maintained under a constant temperature of 22°C at five to six animals per cage (Tecniplast Cages 1284L). Animals were fed with *ad libitum* access to food and water as in compliance with European standards for the care, and use of laboratory animals and experimentations conducted in this study were authorized by the French Direction of Veterinary Services with the approved registration number APAFIS#10392-201706231206250v4.

### Drug Treatments

PEL24-199 and MAGS02-14 compounds were synthesized as previously described (Gay et al., 2018). A safety pilot study was performed in WT animals treated for one month to establish the innocuousness of compounds MAGS02-14 and PEL24-199 at a dose of 1 and 5 mg/kg. Following the treatment of WT animals, PEL 24-199 and MAGS02-14 were measured in the brain tissue (Supplementary Figure S1). For the present study, animals (*n* = 10 per condition) were randomly distributed, and THY-Tau22 and WT mice were treated for 6 weeks, starting at 6 months of age. MAGS02-14 or PEL24-199 treatment was delivered in the drinking water at a final concentration of 1 mg/kg, that is, 12.5 μg/ml for drinking solutions considering an average weight of 25 g/mouse drinking 4 ml per day. Drinking bottles were changed once every week as aqueous solutions of compounds MAGS02-14 and PEL24-99 were previously demonstrated to be stable during more than 1 week. The volume of solution consumed by the mice was measured throughout the treatment period.

### Behavioral Tests

#### Anxiety

All behavioral procedures were performed blind to the treatment administered. Anxiety, which could interfere with a memory test, was assessed in treated and untreated animals using the elevated plus maze test (EPM). Mice were placed in the center of a plus-shaped maze consisting of two 10 -cm-wide open arms and two 10-cm-wide enclosed arms elevated at 50 cm above the floor. Parameters including distance moved, velocity, the number of entries into each arm, time spent in the open vs. the closed arms, and percentage of open arms entries were acquired during 5 min by video recording using EthoVision video tracking equipment and software (Noldus Information Technology, Paris, France) in a dedicated room.

#### Short-Term Spatial Memory

Short-term spatial memory was assessed using the Y-maze task. The Y-maze task consists of three 10 -cm-wide enclosed arms surrounded by four spatial cues. One of the two arms opposite to the starting (S) arm was alternatively closed during the learning phase. Each mouse (*n* = 10 mice per group) was positioned in the starting arm and was free to explore the maze for 5 min. Then during the retention phase of 2 min, the mouse was returned to the home cage. During the test phase of 5 min, the closed arm was opened, and the mouse was placed in the starting arm. The closed arm was then named the “New arm” (N), and the two other arms were named “Others” (O). Parameters—total distance traveled, velocity, the alternation between the arms, and entries into the three arms—were measured during 5 min. The short-term spatial memory test was considered successful when the proportion of entries in the new arm was significantly higher than the time spent in the other two arms during the first 2 min of the test.

### Sacrifice and Brain Tissue Preparation

The mice were sacrificed by beheading in order to prevent an influence of anesthetization (Le Freche et al., 2012). The blood was collected from the neck in heparinized tubes. For immunohistochemistry, one hemibrain was immersed in 4% paraformaldehyde in PBS (pH 7.4) for a week at 4°C and transferred to 20% sucrose solution overnight before being frozen. Cortex and hippocampus of the other half of the brain were dissected, with each split in 1.5 ml isopropylene tubes, and snap-frozen by immersion of the tubes in isopropanol solution added with dry ice. Brain tissues were then stored at −80°C until biochemical analyses. For biochemical analyses, cortex and hippocampus were thawed on ice, and were then added with a volume of ice-cooled Tris-sucrose buffer (TSB) (Tris-HCl 410 mM, pH 7.4 added with 10% sucrose) to reach a final volume of 200 µl. Brain tissue homogenates were further sonicated (40 pulses of 0.5 ss, amplitude 40%, 20 kHz) on ice. Protein concentrations were determined using the BCA Protein Dosage Kit (BioRad, France).

### Insoluble Tau Fraction Preparation

Brain tissue homogenates in TSB buffer (crude) were centrifuged at 14,000 rpm for 10 min (Centrifuge 5424R, Eppendorf). The supernatant (S1) was added with TSB to a final volume of 600 µl and sonicated (40 pulses of 0.5 s, amplitude 40%, 20 kHz). The brain tissue homogenates were then spun at 49, 000 rpm for 1 h (Optima TLX ultracentrifuge equipped with a TLA-110 rotor, Beckman). The supernatant was collected, and a pellet was resuspended in 600 µl of a Tris-Triton (2%) solution (Tris-HCl 10 mM pH 7.4, 2% Triton X-100) (S2). The S2 samples were sonicated and spun at 49, 000 rpm for 1 h. The resulting S3 supernatant was recovered, and the pellet (C3) was resuspended in one volume of NuPAGE™ LDS 2X Sample Buffer supplemented with NuPAGE™ Sample Reducing Agent (10x) (Invitrogen), following the manufacturer’s instructions. The NUPAGE™ Western blot protocol was applied, and 8 μL of crude, 10 μL of S1, 15 μL of S2 and S3, and 20 μL of C3 were loaded per well. Western blot signals were acquired using the LAS-3000 (Fuji), and protein expression levels were determined using ImageQuantTL software. Results (*n* = 4 per group of animals) were expressed as the ratio of the protein in the insoluble fraction divided by the protein signal detected in the soluble fraction plus that measured in the insoluble fraction.

### Bioavailability Assessment

#### Analyte Mouse Brain Extraction

Fifty mg of brain tissue (2 mice per group) were thawed in a safe lock microtube with 500 µl of 1% HCl with one 5 mm tungsten carbide bead. The microtubes were loaded in the TissueLyser II (Qiagen) support plates (24 × 2) at 80°C during 2 × 5 min at 25 Hz (between two cycles, 180 plate rotation). The tubes were centrifuged at 12,000 rpm (Centrifuge 5424R, Eppendorf) for 10 min at 4°C. The supernatant (200 µl) was placed in a polypropylene tube, and 1800 µl of acetonitrile containing the internal standard (Verapamil 1 nM) at −20°C was added. Each tube was stirred for 30 s and placed for 1 h at −20°C for protein precipitation. The tubes were centrifuged at 4,000 tr/min (Centrifuge 5424R, Eppendorf) for 10 min at 4°C. 1.8 ml from each tube was withdrawn and transferred to another tube for evaporation using the Genevac™ centrifugal evaporator for 4 h at 30°C. The residue was dissolved with 200 µl of acetonitrile, vigorously stirred, and evaporated in Genevac™ centrifugal evaporator for 1 h at 30°C. The final residue was dissolved with 90 µl of methanol, vigorously stirred, filtrated, and placed in a Matrix tube for mass spectrometry.

#### Analytical Equipment

LC-MS/MS analysis was performed with an Acquity UPLC–MS Waters I-Class coupled to a Xevo TQS Mass Spectrometer (Waters^®^). Instrument control, data acquisition, and processing were made by MassLynx™ software, and the reprocessing was carried out using MassLynx™ sub-software (TargetLynx). The separation was carried out on a Waters^®^ Acquity BEH [C18, 50 × 2.1 mm, 1.7 µm (40°C)]. 1 µl of the sample was injected, and elution was performed at a constant flow rate of 500 μL/min with H_2_O-ammonium formate 5 mM (pH 3.75) as eluent A and acetonitrile-ammonium formate (5 mM, 5% H_2_O) as eluent B, employing a 0.1-min step at 2% B and a linear gradient from 2% B to 98% B in 1.9 min, followed by a 0.5 min step at 98% B. Then, column re-equilibration was achieved after 1.5 min. MS analysis was carried out in positive ionization mode using an ion spray voltage of 5000 V. The nebulizer (air) and the curtain (argon) gas flows were set at 0.5 bar. The source temperature and the cone gas flow were set at 150°C and 50 L/h, respectively. The desolvation temperature and desolvation gas flow were set at 600°C and 1200 L/h, respectively. The multiple reaction monitoring (MRM) transitions were monitored with the following values: PEL24-199: 478.40/125.98; MAGS02-14: 479.40/112.04; and Verapamil (internal standard): 455.32/165.04. The collision energies were 42 eV (PEL24-199), 46 eV (MAGS02-14), and 56 eV (Verapamil) for all these transitions.

### SDS-PAGE and Western Blot

Hippocampus and cortex samples were prepared at a final concentration of 1 mg/ml of total brain lysate protein in TSB with NuPAGE™ LDS 2X Sample Buffer supplemented with NuPAGE™ Sample Reducing Agent (10x), following the manufacturer’s instructions (Invitrogen). Brain homogenates were then heated for 10 min at 70°C. For each LDS brain lysate, 8 μg of total brain protein were loaded per well onto precast 12% Criterion™ XT Bis-Tris polyacrylamide 26-well gels (Bio-Rad) to analyze tau phosphorylation. The 4–12% Criterion™ XT Bis-Tris polyacrylamide 18-well gels were used for all other Western blot analyses. Criterion™ Cell and the NuPAGE™ MOPS SDS Running Buffer (1X) were used. Electrophoreses were achieved by applying a continued tension of 100 V per gel for 60 min. The apparent molecular weight calibration was determined using molecular weight markers (Novex and Magic Marks, Life Technologies). Following electrophoresis, proteins were transferred to a nitrocellulose membrane of 0.4 μm pore size (GE Healthcare) using the Criterion™ blotting system by applying a continued tension of 100 V for 40 min. Quality of electrophoresis and protein transfer was determined by a reversible Ponceau Red coloration of protein transferred onto the nitrocellulose membrane (0.2% xylidine Ponceau 2R and 3% trichloroacetic acid). After extensive washing under deionized water, membranes were blocked during 1 h in 25 mM Tris–HCl pH 8.0, 150 mM NaCl, 0.1% Tween-20 (v/v) (TBS-T) with 5% (w/v) of skimmed milk (TBS-M), or 5% (w/v) of bovine serum albumin (TBS-BSA) (see Supplementary Table S1). Membranes were then incubated with primary antibodies overnight at 4 °C. Conditions of use of primary and secondary antibodies are summarized in Supplementary Table S1. Membranes were rinsed 3 times for 10 min with TBS-T, and then incubated with secondary antibodies for 45 min at room temperature. The immunoreactive complexes were revealed using either the ECL™ or ECL™ Prime (Cytiva), following the manufacturer’s instructions, and Western blot images and signals were acquired with the LAS-3000 system (Fuji). Quantifications of protein expression were calculated with ImageQuant™ TL software, and values for each sample were divided by the values of GAPDH staining. The semi-quantitative results for samples of the treated conditions were divided by the semi-quantitative values of the control samples to express the results as the percentage of the untreated condition. An average of six to seven mice per group was analyzed for this experiment.

### Two-Dimensional Gel Electrophoresis

Two-dimensional electrophoresis of tau protein was performed as described (Sergeant et al., 2017). Briefly, 15 µg of total brain proteins (pool of five mice for each group) were added with 15 µl of Tris 20 mM containing 2% SDS and heat-treated at 100°C for 5 min. Proteins were then precipitated with 10 volumes of cold acetone (–20°C), incubated at –20°C for 20 min, and centrifuged at 14,000 x g for 10 min. The supernatants were removed, and the protein pellets were left to dry for 1 h at room temperature before being resuspended in 200 µl of IEF buffer (8 M urea, 2 M thiourea, and 4% CHAPS). Samples were sonicated (40 pulses of 0.5 s, amplitude 40%, 20 kHz) on ice, and each tube was added with 2D electrophoresis buffer (1.1 µl of IPG buffer pH 3–11), 2.2 µl of DeStreak Rehydration Solution (Cytiva), and a bromophenol blue trace. Then samples were loaded on 11 cm Cytiva (pH 3–11) IPG strips covered with mineral oil and left to rehydrate passively overnight. The IPG strips were then charged in an Ettan™ IPGphor™ Manifold (Cytiva), and isoelectrofocalization was achieved by applying 0.5 kV for 1 h, 1 kV for 1 h, and 6 kV for 2 h (for each experiment, samples are processed in the same run of IEF). The IPG strips were then equilibrated three times (10 min each) in an equilibration buffer (25 mM Tris-HCl pH 6.8, 20 mM DTT, 10% glycerol, 5% SDS, and 0.05% bromophenol blue) and were layered onto a Criterion XT 4–12% precast gradient Bis-Tris Polyacrylamide Gel. SDS-PAGE was performed according to the regular Western blot protocol. Tau protein isovariants were detected with the Pan anti-tau Cter antibody (Supplementary Table S1).

### Immunohistochemistry and Image Analysis

Coronal free-floating brain sections of 40 µm were obtained with a cryostat (CM3050 S, Leica). The sections of the hippocampus were selected according to the stereological rules and were stored in PBS (phosphate buffer saline) with 0.2% sodium azide at 4°C. For visible phospho-tau and GFAP immunohistochemistry, the coronal brain sections were permeabilized with a 0.2% Triton X-100 solution in PBS. Sections were then incubated with a 0.3% hydrogen peroxide solution and further blocked with 10% “Mouse on Mouse” Kit serum (ZFO513, Vector Laboratories) for 1 h before incubation with primary anti-tau or anti-GFAP antibody overnight at 4°C. Antibodies used in this study are listed in Supplementary Table S1. After washing in PBS, the sections were incubated with biotinylated anti-mouse or anti-rabbit IgG secondary antibody for 1 h. Then sections were incubated with the ABC Kit (Vector Laboratories) for 2 h and developed using DAB (Sigma) before being rinsed with a physiological solution. Brain sections were mounted on glass slides (Superfrost Plus, ThermoScientific) and dehydrated by sequential baths in 30, 70, 95, and 100% ethanol for 5 min. Then the slides were immersed in toluene for 15 min and fixed with mounting medium (VectaMount Permanent Mounting Medium H-5000, Vector Laboratories) and glass coverslips. Images were acquired using Zeiss Axioscan. Z1 slidescan, and quantification of the NFT-containing neurons was performed by counting the number of events in the CA1 area of the hippocampus, for three anteroposterior sections in mean selected according to the Allen mouse brain atlas in an average of four mice for each group.

For immunofluorescence studies, coronal brain sections were permeabilized with an 0.2% Triton X-100 solution in PBS and blocked with normal goat serum (1/100; S1000 Vector Laboratories) in PBS for 1 h before incubation with an anti-GFAP antibody and anti-_S_422 at 4°C overnight. After washes, sections were incubated with secondary antibody AlexaFluor 568 goat anti-mouse IgG and AlexaFluor 488 goat anti-rabbit IgG in 0.2% Triton X-100 in PBS for 1 h. Sections were incubated with DAPI (1/5000; Sigma-Aldrich) for 5 min and mounted on glass slides. Sections were finally treated with 0.3% Suden Black (Millipore 2,160) for 5 min and washed with 70% ethanol to block autofluorescence. Images were acquired using a Fluorescence Zeiss Axioscan. Z1 slidescan. Quantification of GFAP staining and _S_422 were performed using NIH ImageJ software and a custom macro. The number of NFT-containing neurons marked by _S_422 phospho-tau antibody and the surface of GFAP staining is expressed as a ratio over the section brain surface (in mm^2^) analyzed, and was determined in the hippocampus of THY-Tau22 mice.

### ELISA Measurements

The blood samples in heparinized tubes were centrifuged at 10,000 rpm for 15 min (Centrifuge 5424R, Eppendorf), and the plasma was recovered. Plasma levels of total human tau protein were obtained using an ELISA kit (Total Tau ELISA, EuroImmun, EQ6531-9601-L), following the manufacturer’s instructions. Briefly, 100 µl of biotin solution per well were incubated with 25 µl of samples, calibrators, and controls during 3 h at room temperature. The ELISA plate was washed using the washing buffer, and 100 µl per well of enzyme conjugate was added for 30 min. The wells were washed again, and 100 µl per well of chromogen/substrate were incubated for 30 min protected from light. 100 µl of stop solution were added per well, and the absorbance at 450 nm was measured with Multiskan Ascent spectrophotometer plate reader (ThermoLab Systems). The amounts of total tau in the plasma were assessed in six mice per condition by referring to the standard curve of the manufacturer and expressed in pg/mL.

### Statistics

Results are expressed as means ± SEM. Differences between mean values were determined using the Student’s *t*-test or a Mann–Whitney U-test using GraphPad Prism software 8.4.2. *p* values < 0.05 were considered significant.

## Results

### PEL24-199 Treatment Restores the Short-Term Memory Deficits in a Mouse Model of Tau Pathology

**FIGURE 1:**
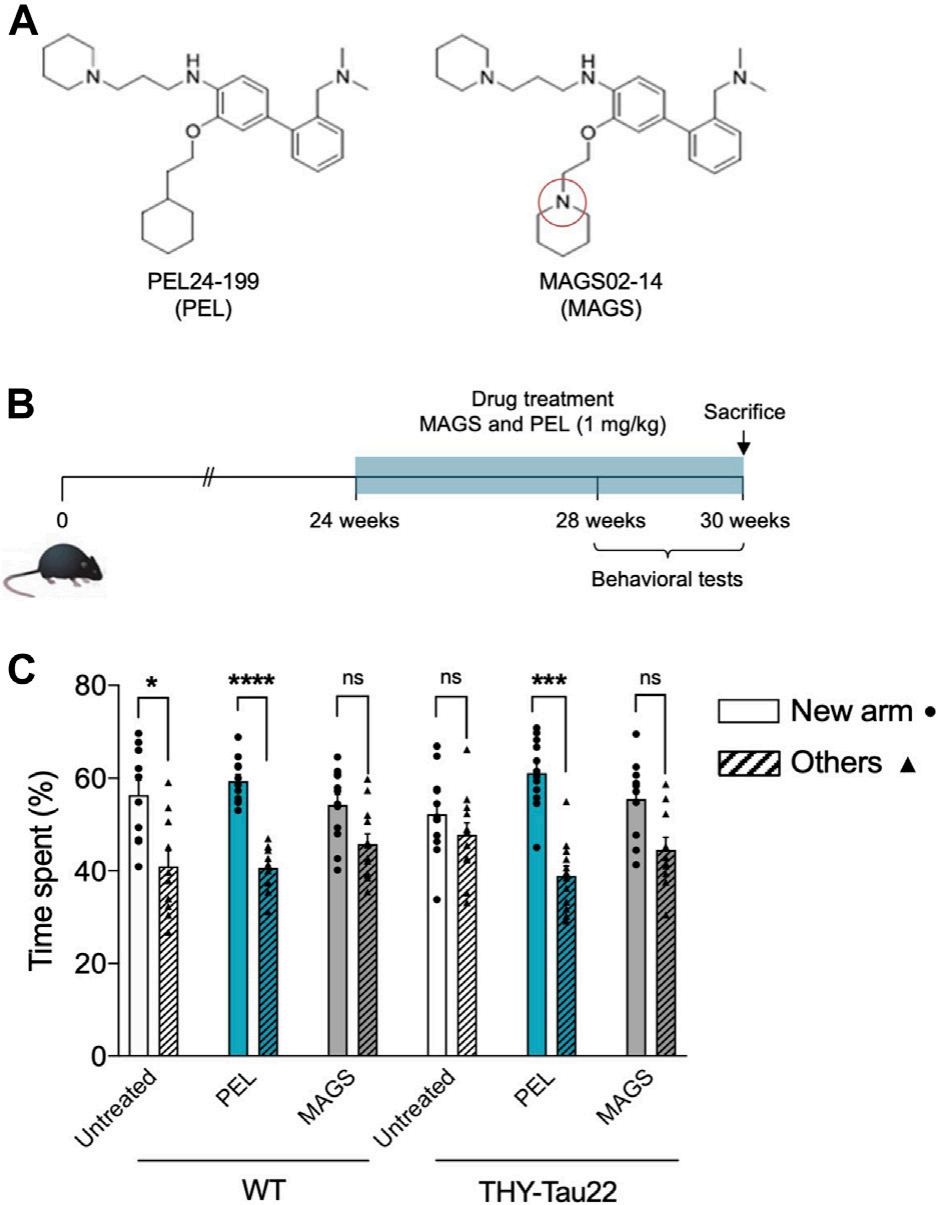
PEL24-199 restores short-term memory in a mouse model of tau pathology. **(A)** Chemical structure of PEL24-199 (PEL) and MAGS02-14 (MAGS) compounds which differ by one nitrogen atom (red circle). **(B)** Time representation of THY-Tau22 mice treatment and behavioral testing. The mice were treated from week 24 to 30 with 1 mg/kg of PEL24-199 or 1 mg/kg of MAGS02-14. **(C)** Short-term memory test of untreated (uncolored) vs. PEL24-199 (blue) and MAGS02-14 (gray)–treated THY-Tau22 animals. Histograms represent the means ± SEM (*n* = 10 animals per condition) of the time spent in the new arm (dots) vs. the other two arms (triangles and hatched bars). Significance at the Mann–Whitney statistical test is indicated by *: *p* < 0.05, **: *p* < 0.01, ***: *p* < 0.001, and ****: *p* < 0.0001. The standard error of the mean is indicated at the top of each histogram bar.

Although NFTs are observed in the hippocampus of THY-Tau22, cognitive impairment appears to be moderate before 6 months of age (Carvalho et al., 2019). The pathology strengthens at 7 months, a stage at which THY-Tau22 mice exhibit spatial memory impairments and ongoing tau pathology development (Sergeant et al., 2019). The associated spatial memory deficits then worsen over time to reach a maximum of 10 months (Schindowski et al., 2006; Van der Jeugd et al., 2013). In order to compare the *in vivo* effects of PEL24-199 and MAGS02-14 (Figure 1A), global behavioral and short-term spatial memory tests were carried out at 7 months of age, following the 6 weeks of treatment in a curative paradigm (Figure 1B). The anxiety measured using the elevated plus maze test showed no significant impact of PEL24-199 and MAGS02-14 treatments on velocity, average distance moved, or percentage of time spent in the closed or open arms for either WT or THY-Tau22 mice (*n* = 10 animals per group; *p* = 0.53, Supplementary Figure S2). Thus, the treatments did not significantly affect the basal anxiety behavior of both WT and THY-Tau22 mice, suggesting that short-term spatial memory assays following the treatments were not influenced by cognitive deficits. In the short-memory Y-maze task, 7 months WT mice treated at 1 mg/kg with MAGS02-14 spent less time in the new arm than the untreated WT mice (Figure 1C). At the same dose, PEL24-199 did not alter the performance of the WT mice. At 7 months of age, THY-Tau22 mice exhibited a short-term spatial memory impairment with an absence of preference between the new arm and the others. MAGS02-14 treatment had no significant effect on the spatial memory of THY-Tau22 mice. In contrast, PEL24-199 mitigated memory impairments of THY-Tau22 mice (Figure 1C).

### PEL24-199 Decreases Hyperphosphorylated Tau in Mice Brain Extracts

**FIGURE 2:**
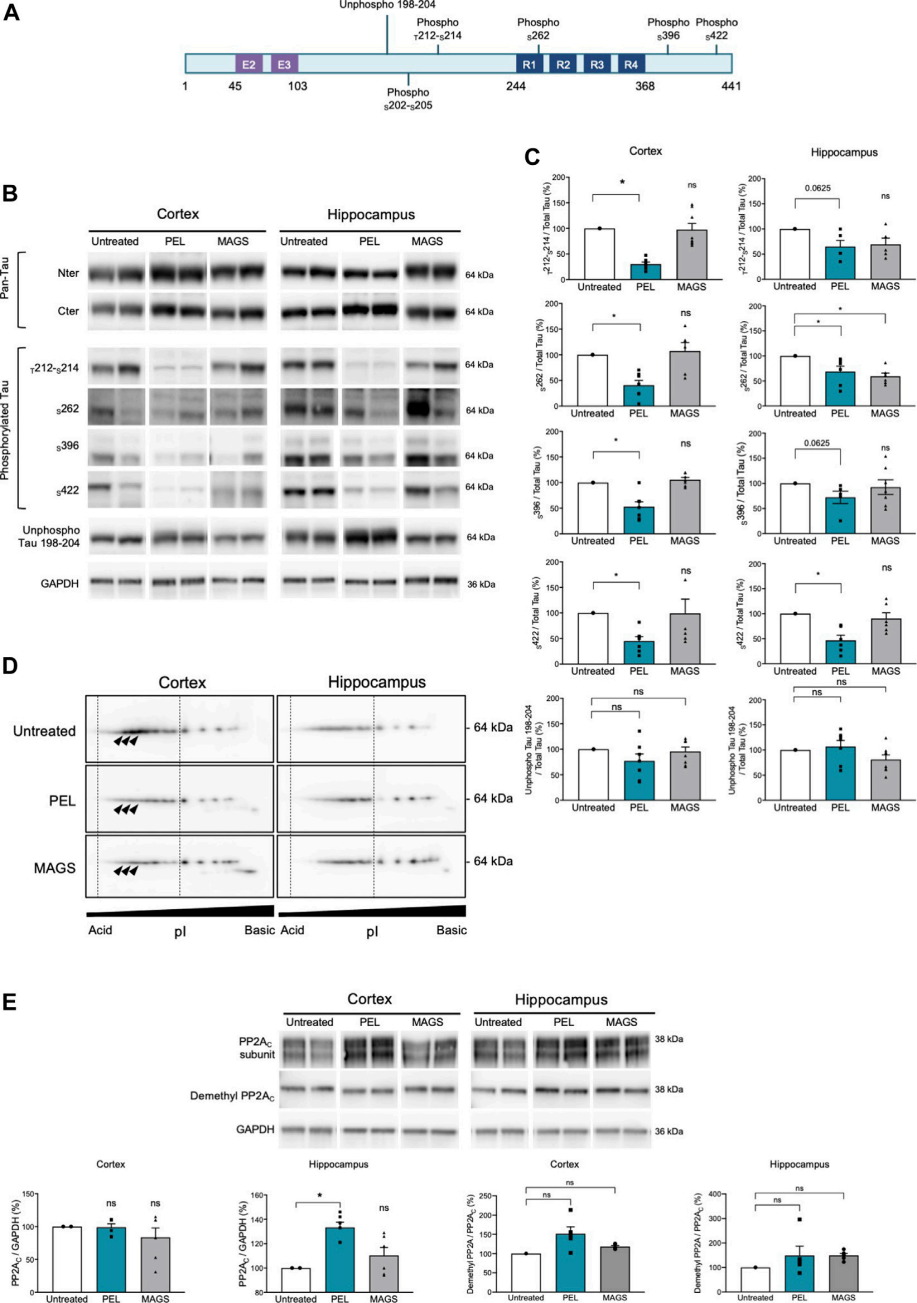
PEL24-199 decreases hyper and pathological tau phosphorylation and increases PP2A_C_ expression. **(A)** Positioning of unphospho- or phosphorylated epitopes recognized by tau antibodies (numbering according to the longest human brain tau isoform of 441 amino acids). Exon two and exon three encoding sequence and the microtubule-binding region formed by tau repeated sequences numbered R1 to R4 are represented. **(B)** Western blotting of tau expression using N-terminus (Nter) and C-terminus (Cter) pan-Tau antibodies, and phosphorylated tau at threonine 212 and serine 214 (_T_212-_S_214), serine 262 (_S_262), serine 396 (_S_396), serine 422 (_S_422), and unphosphorylated tau residues comprised in tau sequence 198-204 (unphospho Tau 198-204). Antibodies labeling are presented for untreated, PEL, and MAGS-treated animals for protein extracts from the cortex or the hippocampus. **(C)** Histogram representations of the % of antibodies labeling. The control percentages (untreated conditions, uncolored bars) were given the value of 100%. PEL (blue bars) and MAGS (gray bars) means ± SEM percentages to the control value are represented. Significance at the Mann–Whitney test is indicated *: *p* < 0.05. **(D)** Two-dimensional gel electrophoresis and Western blotting of human tau isovariants from the cortex or hippocampus from untreated, PEL, or MAGS –treated animals. Acido-basic (pI) orientation of 2D Western blots is indicated on the *x*-axis, and tau protein apparent molecular weight of 64 kDa is indicated on the *y*-axis. The vertical dotted lines encompass the most acidic tau isovariant, and differences are indicated by arrowheads. Note that tau isovariants extend toward more acidic isoelectric points in hippocampal THY-Tau22 brain extracts from untreated animals. Two-dimensional electrophoresis is representative of a pool of five animals per condition. **(E)** Western blots of PP2A catalytic subunit C (PP2A_C_), demethyl PP2A_C,_ and GAPDH, and histogram representations of the semiquantitative analysis of phosphatase PP2A_C_ catalytic subunit in cortex and hippocampus of untreated (value of 100%), PEL, and MAGS-treated THY-Tau22 animals. Histograms of the ratio of demethyl PP2A_C_ upon total PP2A_C_ expression are expressed as the percentage of the untreated condition which was given the value of 100%.

Cognitive impairment is associated with a progression of the tau pathology in the hippocampus and the cortex of THY-Tau22 mice (Van der Jeugd et al., 2013). Thus, memory impairment could therefore be related to a modification of the tau pathology and tau phosphorylation status. We, assessed the hippocampal and cortical tau expression as well as the tau phosphorylation levels using antibodies raised against N- and C-terminus of tau proteins, and specific phospho-sites are known to be hyperphosphorylated in AD (Augustinack et al., 2002; for a review, see Sergeant et al., 2008) as well as pathological epitopes which are only detected when neurofibrillary processes are present (_T_212/_S_214 and _S_422) (antibody epitopes are represented on Figure 2A). Treatment with PEL24-199 or MAGS02-14 did not change the global expression of total tau proteins either in the hippocampus cortex of THY-Tau22 mice (Figure 2B, pan-Tau antibodies). Tau phosphorylation at either physiologic or pathological epitopes was not significantly diminished, except for phosphorylation at serine 262 (Figure 2C) following MAGS02-14 treatment (Figures 2B,C). Noticeably, PEL24-199 decreased the level of phosphorylated tau at _S_396 and _S_262 and significantly diminished the labeling of pathological epitopes _T_212/_S_214 and _S_422 in the cortex by half (Figures 2B,C). Further, PEL24-199 significantly reduced the phosphorylation of tau at _S_262, _S_396, and _S_422 sites, and _T_212/_S_214 phospho-sites; however, it was not statistically significant (*p* = 0.0625) in the hippocampus of THY-Tau22–treated mice (Figure 2C). Levels of unphosphorylated tau at 198-204 amino acid sequence did not change under PEL24-199 or MAGS treatments. Global phosphorylation status was then analyzed by 2D gel electrophoresis and labeling of tau with the pan-Tau Cter antibody. Phosphorylated isovariants are resolved toward the acidic isoelectric points of 2D gels. Reduced intensity of those acidic isovariants was observed in the cortex and hippocampus of THY-Tau22 mice treated with PEL24-199 and MAGS02-14 when compared to untreated THY-Tau22 mice (Figure 2D, isovariants comprised the dotted lines and were indicated by arrowheads). As tau phosphorylation is controlled by phosphatases and kinases, expression of the principal tau serine/threonine phosphatase PP2A (Liu et al., 2005) was investigated. On treatment with MAGS02-14 (Figure 2E) as well as in the cortex for PEL24-199–treated mice (Figure 2E), the catalytic subunit PP2A_C_ expression remained unchanged. However, we noticed a sharp increase of PP2A_C_ expression in the hippocampus of THY-Tau22 mice treated with PEL24-199 (Figure 2E). Methylation or demethylation of the catalytic subunit of PP2A_C_ at leucine 309 residue reflects its phosphatase activity, where methylated PP2A_c_ corresponds to the activated form of the phosphatase (Papon et al., 2013; Sontag and Sontag 2014; Ahmed et al., 2020). The ratio of demethylated PP2A_C_ showed no significant modification between untreated and treated animals, suggesting an unchanged activity (Figure 2E).

### PEL24-199 Decreases Detergent-Resistant Phospho-Tau in Mice Hippocampus

**FIGURE 3:**
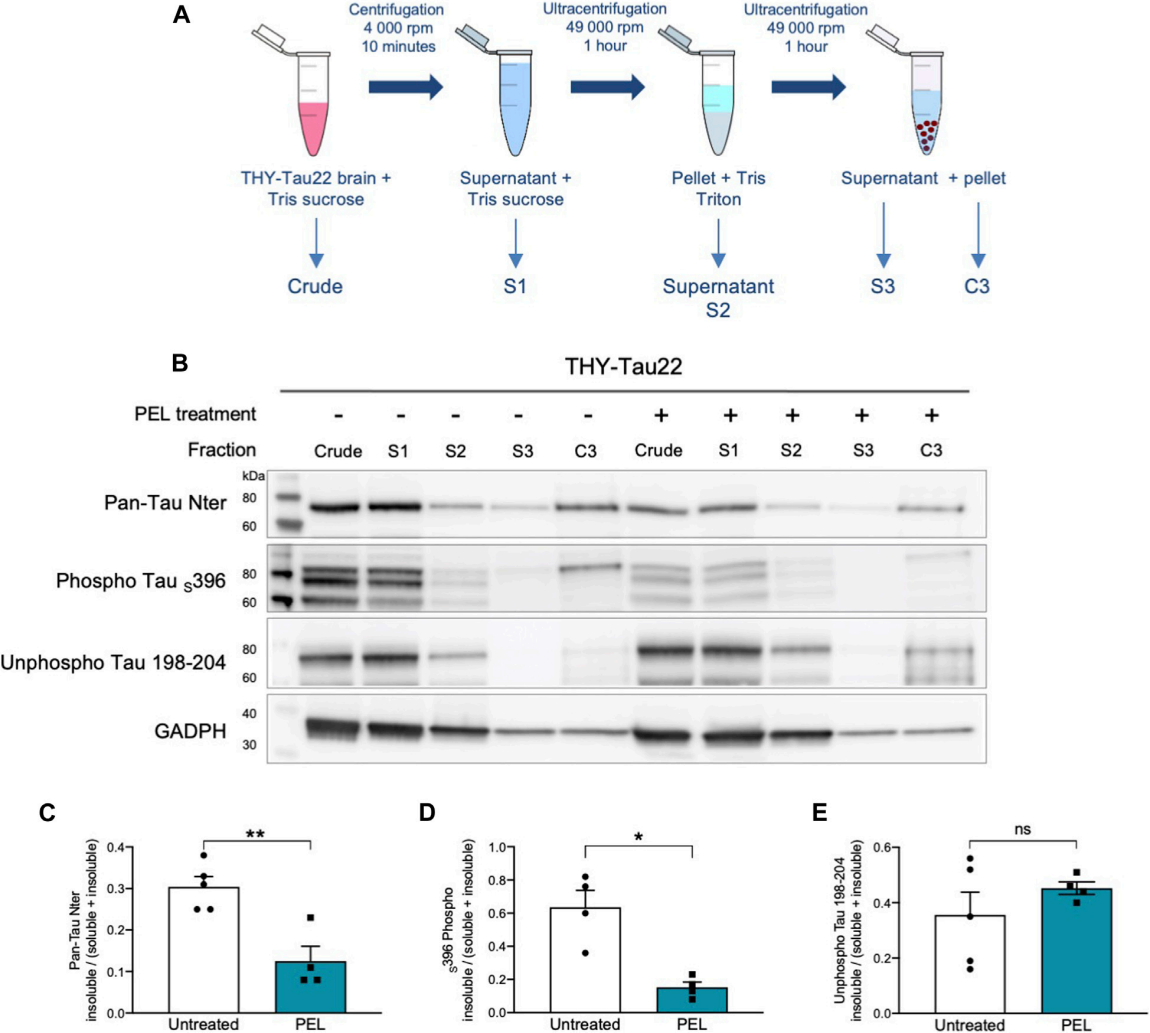
PEL24-199 treatment decreases the insoluble phosphorylated tau fraction in THY-Tau22 mice. **(A)** Schematic representation of mouse brain tissue fractionation steps. The samples in TSB (Crude) are centrifuged, and the supernatant is added with 10% TSB (S1), and sonicated before being centrifuged at 100,000 ×g. The supernatant is collected (S2), and the pellet is resuspended in Tris-Triton X-100 buffer, sonicated, and spun at 100,000 ×g for 1 h. The supernatant (S3) is collected, and the pellet is resuspended in SDS-PAGE LDS lysis buffer (C3). Each of the fraction from S1 to C3 and the crude brain lysate are loaded onto SDS-PAGE. **(B)** Western blot of tau proteins (Pan-Tau Nter), phosphorylated tau proteins at serine 396 (Phospho Tau _S_396), and unphosphorylated tau proteins (Unphospho Tau 198-204) in crude, S1 to S3 and insoluble C3 pellets from the hippocampus of THY-Tau22–untreated (−) or PEL-treated (+) mice. GAPDH staining was performed to ascertain that an equal quantity of material was loaded between untreated and treated protein fractions. **(C,D, E)** Histogram representations of the mean ± SEM ratio between the signal of the insoluble fraction divided by the signal in the soluble plus insoluble fraction for Pan-Tau Nter, phospho Tau _S_396, and unphosphorylated Tau 198-04 antibodies labeling in the hippocampus fractions S1 (soluble) and insoluble (C3) of THY-Tau22 treated or not with PEL24-199 (*n* = 5 for untreated and *n* = 4 for PEL treated animals). The mean difference was statistically considered as significant with a *p*-value below 0.05 (indicated by one asterisk).

NFTs are characterized by tau aggregation of hyper- and abnormally phosphorylated tau proteins. This aggregation is associated with an increased insolubility of tau (Schindowski et al., 2006). Tau solubility was further investigated in soluble and insoluble tau fractions (Sergeant et al., 2003) from THY-Tau22 mice treated with either MAGS02-14 or PEL24-199. MAGS02-14 treatment unchanged the distribution of tau or phosphorylated tau at serine 396 either in soluble or insoluble protein fractions (Supplementary Figure S3), whereas total tau and phosphorylated tau at serin 396 were reduced in most insoluble C3 fraction from brain tissue of mice treated with PEL24-199 (Figures 3B–D). The unphosphorylated tau proteins remained unchanged in this same insoluble fraction (C3) (Figures 3B–E). Therefore, while MAGS02-14 at 1 mg/ml did not affect tau insolubility, PEL24-199 diminished both total tau and phospho-Tau insolubility in THY-Tau22–treated animals.

### PEL24-199 Reduces NFTs and Astrogliosis in the Hippocampus of THY-Tau22 Treated Animals

**FIGURE 4:**
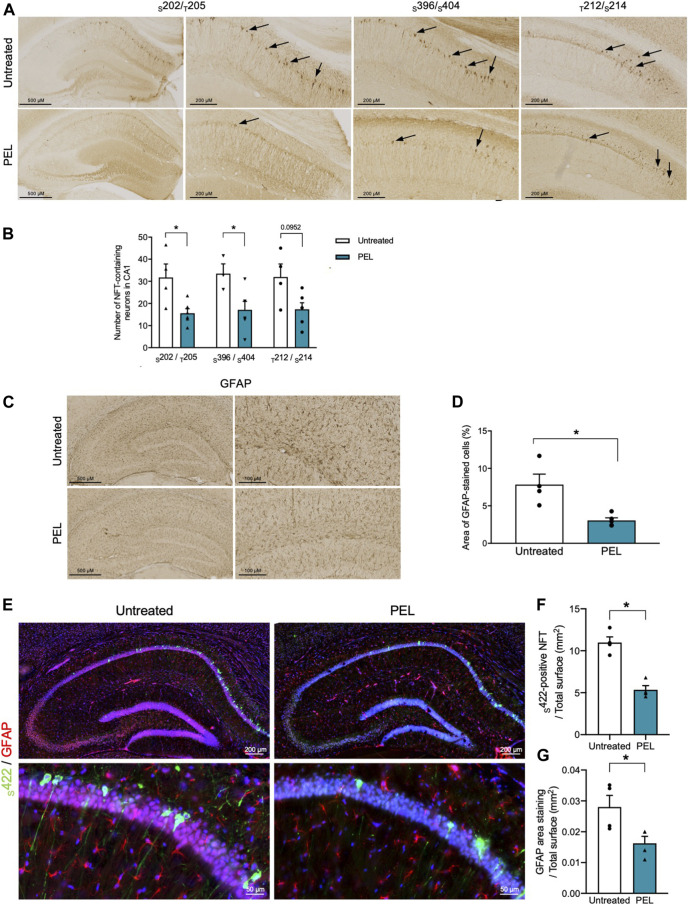
Tau pathology and astrogliosis are reduced in PEL24-199 THY-Tau22–treated animals. **(A)** Immunohistochemistry with the phospho-Tau antibodies against phospho-epitopes _S_202/_T_205 and _S_396/_S_404 or pathological epitopes (_T_212/_S_214) in the hippocampus of THY-Tau22 animals treated or not with PEL24-199 (PEL). **(B)** The mean ± SEM number of NFT-labeled neurons observed in three brain slices per animal (*n* = 4 animals per condition) are represented on the histogram. Labeled neurons with the _S_202/_T_205, _S_396/_S_404, and _T_212/_S_214 were counted in the hippocampus of untreated (uncolored bars) and PEL24–199–treated (blue bars) animals. Statistical differences between untreated and treated conditions were significant with a *p*-value below 0.05 (*). **(C)** Reactive astrocytes were labeled with glial fibrillary acidic antibody (GFAP) in sections of the hippocampus of untreated and PEL24-199 (PEL)–treated THY-Tau22 mice. **(D)** Histogram representation of the mean ± SEM ratio of GFAP labeling upon the total hippocampal surface in mm^2^ (three individual hippocampal sections per animal and a minimum of four animals per condition). Statistical differences between untreated and treated conditions were significant with a *p*-value below 0.05 (*). **(E)** Immunofluorescence analysis of _S_422-positive NFTs and astrogliosis in the hippocampus of THY-Tau22 mice treated or not with PEL24-199 (PEL). The _S_422 antibody was used for the detection of NFTs (in green) and the GFAP antibody was used to stain reactive astrocytes (in red). Note that the _S_422 and GFAP staining were significantly reduced in THY-Tau22 mice treated with PEL24–199 (PEL). **(F)** Ratio of the number of _S_422-positive NFTs over the total surface in mm^2^ of the CA1 of the hippocampus of untreated or PEL24–199 (PEL) THY-Tau22–treated animals. Statistical differences between untreated and treated conditions were significant with a *p*-value below 0.05 (*). **(G)** Ratio of the glial fibrillary acidic protein (GFAP) staining over the total surface in mm^2^ of the hippocampal CA1 of PEL24-199 (PEL) treated or untreated THY-Tau22 mice. Statistical differences between untreated and treated conditions were significant with a *p*-value below 0.05 (*). Results are expressed as the mean ± SEM from three brain slices of four animals per condition.

THY-Tau22 mice exhibit neurofibrillary tangles as well as mild astrogliosis (Schindowski et al., 2006). To further assess the modulatory effect of our compounds, the burden of NFTs in the hippocampal CA1 was investigated by immunohistochemistry using antibodies against hyperphosphorylated tau epitopes _S_202/_T_205 and _S_396/404 and pathological tau phospho-sites _T_212/_S_214 (Figure 4A). Treatment with MAGS02-14 resulted in a slight, although not significant, reduction in the number of NFTs. (Supplementary Figure S4A). In PEL24-199 THY-Tau22–treated animals, _S_202/_T_205 and _S_396/_S_404-positive NFTs were significantly reduced (Figure 4B). The number of NFTs stained with pathological _T_212/_S_214 phospho-Tau antibody was reduced, although not significantly (*p* = 0.0952). Noticeably, we observed a significant reduction of NFTs stained by the _S_422 phospho-Tau antibody by immunofluorescence, further demonstrating that PEL24–199 treatment reduced the tau pathology (Figure 4F).

Astroglial activation is activated with the development of tau pathology (Schindowski et al., 2006; Laurent et al., 2017; Laurent et al., 2018) and presumably favors the development of tau pathology (Laurent et al., 2018; Ising et al., 2019). We therefore further investigated the impact of PEL24-199 and MAGS02-14 treatment on astrocytes activation, both by immunohistochemistry and immunofluorescence. MAGS02-14 treatment did not modify the GFAP-positive astrocytes as well as the number of _S_422-positive NFTs (Supplementary Figures S4C–E). THY-Tau22 PEL24-199–treated animals showed a significant reduction of GFAP-positive astrocytes both by immunohistochemistry and immunofluorescence (Figure 4G). This decrease in GFAP staining was associated with limited modification of connexin43 expression, a major gap junction protein of astrocytes (Supplementary Figure S5).

### PEL24-199 or MAGS02-14 Does Not Modify Autophagy in THY-Tau22–Treated Animals

*In vitro*, MAGS02-14 but not PEL24-199 was shown to increase the expression of autophagy markers such as p62 or LC3, whereas both molecules are non-competitive inhibitors of the ß-secretase. We, therefore, analyzed the expression of APP and BACE1 as well as several markers of autophagia. APP and BACE1 expression were not modified by either PEL24-199 or MAGS02-14 treatments (Supplementary Figure S6). Expression of LC3 or p62 remains unchanged in treated conditions as compared to the untreated THY-Tau22 mice (Supplementary Figure S7). Moreover, both mTOR and its downstream target p70S6 kinase expression and phosphorylation (p70S6K) were not modified consequently to MAGS02-14 or PEL24-199 treatments of THY-Tau22 animals, together suggesting that autophagy was not part of the signaling cascade modulated by our drugs.

## Discussion

In the present study, we show that the ß-secretase noncompetitive inhibitor compound PEL24-199 represses the tau pathology, increases PP2A_C_ expression, reduces the GFAP-positive astrogliosis, and improves short-term spatial memory in the well-characterized THY-Tau22 transgenic model of hippocampal neurodegeneration. These results, therefore, suggest that this APP metabolism regulatory compound PEL24-199 mitigates the tau pathology *in vivo*. This effect is observed in a curative paradigm, and results are in line with previous studies, where molecules derived from the same pharmacophore were effective in a preventive paradigm against both amyloid and tau pathologies (Sergeant et al., 2019).

A compound containing the same pharmacophore which is additionally fused to a tacrine moiety, RPEL, was shown to reduce both the amyloid pathology in the APPxPS1 transgenic animals and tau pathology in the THY-Tau22 hippocampal neurofibrillary degeneration model (Sergeant et al., 2019). These effects were also associated with a cognitive improvement, however, in a preventive paradigm since animals were treated starting from the age of 3 months before the appearance of lesions in both transgenic models. A structure–activity relationship strategy was used to compare two compounds that differ by a single nitrogen atom that share a ß-secretase noncompetitive inhibitory effect; however, the lysosomotropic activity was only associated with MAGS02-14 *in vitro* (Gay et al., 2018). This lysosomotropic activity is common to several compounds that were originally derived from chloroquine (Melnyk et al., 2015). Through the alkalization of intravesicular pH, the lysosomotropic activity of compounds inhibits the ß-secretase pH-dependent activity and represses the autophagic flux (Schrader-Fischer and Paganetti 1996; Tam et al., 2014). Dosage of MAGS02-14 in the brain tissue showed an accumulation when compared to PEL24-199 (Supplementary Figure S1). This accumulation could be a contributing factor to its inefficacy and could potentially be deleterious. Modulation of the γ-secretase that is routed to the early endosome together with the ß-secretase is likely not contributing to the observed effect of our compounds since Notch1 γ-secretase processing is not modified by RPEL, MAGS02-14, or PEL24-199 (Gay et al., 2018; Sergeant et al., 2019). Moreover, chloroquine and molecules having a lysosomotropic activity inhibit the autophagy flux, the effect of which was also shown previously *in vitro* for MAGS02-14 (Gay et al., 2018). Several markers of autophagia including mTOR and its downstream target p70S6K (Lipton and Sahin, 2014) as well as p62 and LC3 were analyzed, and no modulation was observed *in vivo* following 7 weeks of treatments of THY-Tau22. Together, our results suggest that the lysosomotropic activity is not necessary for the *in vivo* activity of our compounds, whereas the ß-secretase noncompetitive inhibitory activity is more likely essential.

Although a direct relationship between ß-secretase aspartyl proteases BACE1 or BACE2 and tau protein expression has not yet been established, a growing body of evidence suggests an interplay between tau protein and the ß-secretase processing of APP. ß-secretase inhibitors or γ-secretase modulators were shown to reduce tau protein expression in control neurons derived from human stem cell–derived excitatory cortical neurons (Moore et al., 2015). Following PEL24-199 treatment of THY-Tau22 mice, tau phosphorylation was reduced at hyperphosphorylated sites and pathological phospho-sites. Moreover, the insoluble fraction of tau as well as the number of neurofibrillary tangles was reduced. Notably, the decrease of tau phosphorylation was not followed by an increase in tau plasmatic clearance (Supplementary Figure S8), suggesting that the positive effects observed with PEL24-199 treatment on the decrease of tau pathology are not related to a change in the plasma clearance of tau protein. Modulation of tau phosphorylation can be attributed to the modification of PP2A expression, as there is an inverse relation between the hyperphosphorylation of tau Ser202/Thr205 and PP2A activity (Kins et al., 2003). Moreover, increased activation of PP2A was shown to contribute to the restoration of cognitive functions in THY-Tau22 mice, also in a curative paradigm (Ahmed et al., 2020). PP2A is inhibited in AD and suggested to contribute to the hyperphosphorylation of tau and the regulation of APP metabolism (Taleski et al., 2021). PP2A catalytic subunit expression is increased in THY-Tau22 mice treated with PEL24-199 but not in mice treated with MAGS02-14, first showing the specific effect of PEL24-199, and second, we can assume a relationship between the reduction of tau phosphorylation and increased expression of PP2A. Moreover, the increased expression was not associated with a change of methylation status of PP2A_C_, therefore suggesting that a gain of PP2A activity is more likely a consequence of an increased expression of PP2A. In PEL24-199–treated mice, the insoluble tau fraction was reduced, indicating that the proportion of aggregated tau is diminished, resulting in the lowering of existing neurofibrillary degenerating processes, the inhibition of this process, or both. These results are strengthened by the significant lowering of the number of neurofibrillary degenerating neurons in the brain of PEL24-199–treated animals. We, therefore, demonstrated that PEL24-199 can decrease the tau pathology *in vivo* by reducing the number of NFTs present in the hippocampus. Together, these results demonstrate a reduction of the neurofibrillary degenerating process in THY-Tau22–treated mice when compared to untreated animals, and therefore PEL24-199 compound reduces the tau pathology in a curative paradigm together with the recovery of the short-term spatial memory. Our results are in line with the article of Moore et al. (Moore et al., 2015), in which they showed that manipulating APP metabolism by ß-secretase inhibition results in a specific decrease in tau protein levels, demonstrating that APP metabolism regulates tau proteostasis. Such modulatory effect of both APP and tau was achieved with an activator of the chaperone-mediated autophagia (CMA), further suggesting another therapeutic route active on both APP and tau pathology (Bourdenx et al., 2021). Our data suggest that modulating the metabolism of APP with small molecules can affect not only tau protein levels but also the neurofibrillary degenerating process, and in turn improve cognitive functions.

Few studies involving ß-secretase inhibitors were shown to reverse or attenuate the behavioral and memory deficits in transgenic mouse models of AD (Imbimbo and Watling, 2019). Research into the therapeutics for neurodegenerative diseases have proposed several different small molecules as candidates either targeting Aβ or tau lesions (Morimoto et al., 2013; Lecoutey et al., 2014; Yahiaoui et al., 2016), including autophagy modulators (Silva et al., 2020; Bourdenx et al., 2021), but to our knowledge, none of them, except the CMA activator (Bourdenx et al., 2021), acts on both the amyloid and tau pathological processes. BACE1 and BACE2 degrade Aβ peptides besides just being the proteases necessary to produce Aβ peptides (Abdul-Hay et al., 2012). Thus, current inhibitors may also affect Aβ degradation through incomplete repression of the aspartyl protease activity of BACE proteases (Liebsch et al., 2019). As PEL24-199 is not a direct enzymatic inhibitor of BACE1, this compound may differently modulate the APP metabolism and therefore potentially preclude the detrimental effect of pure ß-secretase inhibitors.

Astrogliosis is an inflammatory response that potentiates the progression of neurodegenerative diseases and can be considered as a potential therapeutic target (Phillips et al., 2014; Chung et al., 2015). Astrocytes have a discrete regulatory function of synapses and neuronal plasticity, and, for instance, specific reduction of connexin43 in astrocytes reduces the memory impairment in APPxPS1 mice (Ren et al., 2018). Levels of GFAP-reactive astrocytes are closely associated with dementia in AD (Perez-Nievas et al., 2013). More recently, senescent astrocyte accumulation was shown to promote the formation of hyperphosphorylated tau aggregates, and the reduction of the senescent astrocytes prevents PS19 tau transgenic mice from cognitive decline, as well as a decline in tau pathology has been reported (Bussian et al., 2018), which shows a close interplay between the tau pathology and reactive astrogliosis. Herein, we showed that GFAP-positive reactive astrocytes were reduced in THY-Tau22 mice treated with PEL24-199 when compared to untreated mice. This GFAP-reactive astrocyte reduction could either result from the direct effect of PEL24-199 on astrocytes or indirectly related to the reduction of the tau pathology. The suppression of tau expression in the double APP/PS1 × rTg4510 transgenic as well as in the rTg4510 transgenic model of tau pathology reduced the burden of NFTs and the astrogliosis, and a relatively larger proportion in the single rTg4510 (DeVos et al., 2018). These results taken together could suggest that the reduced astrogliosis in the THY-Tau22 treated with PEL24-199 could be attributed in part to a direct effect of PEL24-199. This reduced astrogliosis may also contribute to the cognitive improvement observed in PEL24-199–treated animals.

In the present study, we showed that PEL24-199, but not MAGS02-14, leads to a restoration of cognitive functions and also to a reduction of the tau pathology and associated astrogliosis in the tau pathology transgenic model THY-Tau22. The effect of our molecule relies on a modification of APP processing through a noncompetitive ß-secretase modulation effect and where the lysosomotropic activity is dispensable. Thus, PEL24-199 treatment in the curative paradigm reduces the tau pathology and astrogliosis, and restores short-term memory. Together, these results indicate that we have a molecule efficient on APP metabolism (Gay et al., 2018, Tautou et al., unpublished data) but also on tau pathology *in vivo*. Further investigations will be necessary to elucidate the precise molecular mechanism of action of these molecules which are effective on both amyloid and tau pathology.

## Data Availability

The raw data supporting the conclusions of this article will be made available by the authors, without undue reservation.
