# Supplementary material for: A ß-Secretase Modulator Decreases Tau Pathology and Preserves Short-Term Memory in a Mouse Model of Neurofibrillary Degeneration
Source: Front Pharmacol. 2021 Jun 29;12:679335. doi: 10.3389/fphar.2021.679335 (PMC8276176; doi:10.3389/fphar.2021.679335)
Supplement: Supplementary file 1 [file DataSheet1.PDF]

Supplementary Material

Supplementary Table and Figures

WB– Primary antibodies	Species	Dilution	Saturation	Reference
Pan-Tau Nter	Rabbit	1 : 5000	TNT / 5% milk	Homemade 12-41 1242075
Pan-Tau Cter	Rabbit	1 : 4000	TNT / 5% milk	Homemade 993-S2 Y16L-2P [427-441]
$_S$262	Rabbit	1 : 5000	TNT / 5% BSA	Invitrogen 44750G
$_S$396	Rabbit	1 : 10 000	TNT / 5% BSA	Invitrogen 44752G
$_S$422	Mouse	1 : 1000	TNT / 5% BSA	4BDX-1501
$_T$212-$_S$214 (AT100)	Mouse	1 : 1000	TNT, no saturation	Invitrogen MN1060
Unphospho Tau 198-204 (TAU-1)	Mouse	1 : 2000	TNT / 5% milk	Millipore MAB3420
GAPDH	Rabbit	1 : 50 000	TNT / 5% milk	Sigma G9545
PP2A$_C$ subunit	Rabbit	1 : 1000	TNT / 5% milk	Sigma 07-324
LC3B	Rabbit	1 : 2000	TNT / 5% BSA	Cell Signaling 2775S
SQSTM1/p62	Rabbit	1 : 1000	TNT / 5% milk	Cell Signaling 5114S
Demethyl PP2A$_C$ (4B7)	Mouse	1 : 500	TNT / 5% BSA	SantaCruz sc13601
mTOR (7C10)	Rabbit	1 : 1000	TNT / 5% BSA	Cell Signaling 2983S
Phospho mTOR ($_S$2448)	Rabbit	1 : 1000	TNT / 5% BSA	Cell Signaling 5538S
p70S6K (H-9)	Mouse	1 : 500	TNT / 5% BSA	SantaCruz sc8418
Phospho p70S6K ($_T$389)	Rabbit	1 : 1000	TNT / 5% BSA	Cell Signaling 9205S
BACE1	Rabbit	1 : 5000	TNT / 5% BSA	Merck Millipore AB5940
APP-C17	Rabbit	1 : 7500	TNT / 5% milk	Homemade 100354 2071
Connexin 43	Rabbit	1 : 1000	TNT / 5% milk	Life 710700
WB– Secondary antibodies				
Goat anti-Rabbit	Goat	1 : 5000		Vector PI1000
Goat anti-Mouse	Goat	1 : 50 000		Merck Millipore AP200P
IHC – Primary antibodies				
$_S$202-$_T$205 (AT8)	Mouse	1 : 500		Thermo Scientific MN1020
$_T$212-$_S$214 (AT100)	Mouse	1 : 500		Invitrogen MN1060
$_S$396-$_S$404 (AD2)	Mouse	1 : 4		Homemade
$_S$422	Rabbit	1 : 1000		Homemade 98857
GFAP (visible)	Rabbit	1 : 1000		Dako, Z0334
GFAP (fluorescence)	Mouse	1 : 200		SantaCruz sc33673
IHC – Secondary antibodies				
Goat anti-Mouse IgG biotinylated	Goat	1 : 400		Vector ZF0805
Goat anti-Rabbit IgG biotinylated	Goat	1 : 400		Vector BA-1000
Alexa Fluor 568 goat anti-Mouse	Goat	1 : 1000		Invitrogen A11004
Alexa Fluor 488 goat anti-Rabbit	Goat	1 : 1000		Invitrogen A11008

Supplementary Table 1: List of primary and secondary antibodies used for Western-blotting and Immunohistochemistry. The species in which the antibodies were developed, the dilution, the saturation solution and providers' references are indicated.

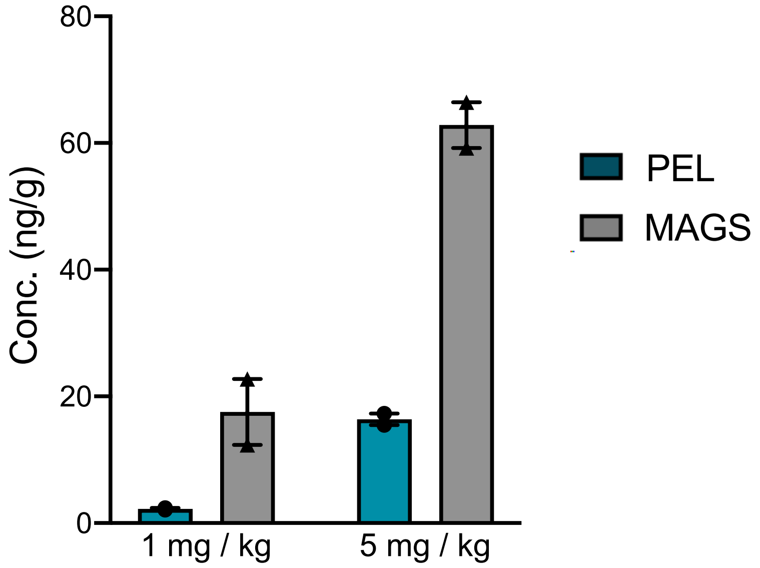

Supplementary Figure 1. Liquid Chromatography and Mass spectrometry quantification of PEL24-199 and MAGS02-14 molecules in the brain of treated animals. PEL24-199 and MAGS02-14 were extracted from brain tissues of WT mice treated with 1 mg / kg or 5mg / kg and quantified by LC - MS/MS TQS procedure. Histograms represent the means ± SEM (n = 2 brains and two-repeated measure for each condition).

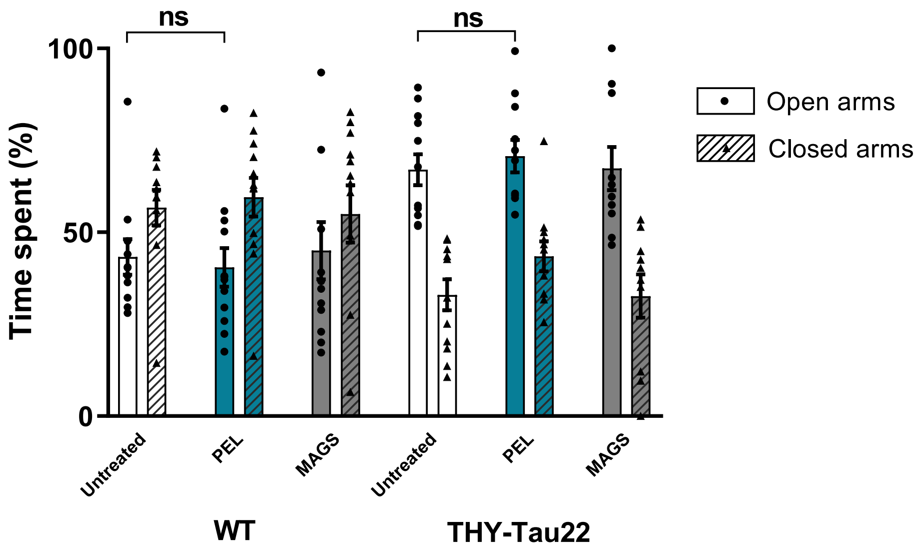

Supplementary Figure 2: Anxiety assessment of THY-Tau22 mice treated or not with PEL24-199 (PEL) or MAGS02-14 (MAGS) drugs. Elevated Plus Maze test data are reported on the histogram as the means ± SEM (n = 10 animals per condition) of the time spent in the open arms (dots) versus the close arms (triangles and hatched bars of untreated (uncolored) versus PEL24-199 (blue) and MAGS02-14 (grey) treated Wild-Type (WT) and THY-Tau22 animals. Note that THY-Tau22 mice are less anxious than Wild-Type mice (WT) but PEL or MAGS treatments had no effect. The standard error of the mean is indicated at the top of each histogram bars. The mean differences were statistically non-significant (ns).

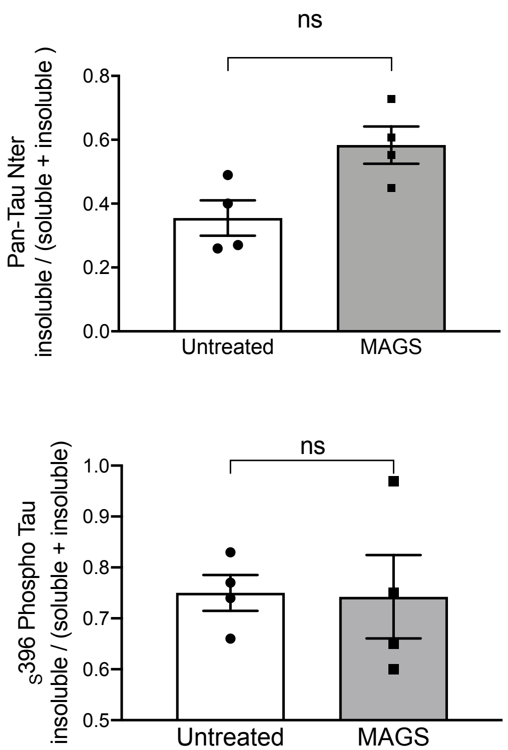

Supplementary Figure 3: Analysis of Tau insolubility in brain extract from THY-Tau22 mice treated with MAGS02-14 (MAGS). Histogram representations of the mean ± SEM ratio between the signal of the insoluble fraction divided by the signal in the soluble plus insoluble fraction for Pan-Tau Nter and Phospho-Tau $_{S}$396 labelling in the hippocampus fractions S1 (soluble) and insoluble (C3) of THY-Tau22 treated or not with MAGS02-14 (n = 5 for untreated and n = 4 for MAGS treated animals). The mean differences were statistically non-significant (ns).

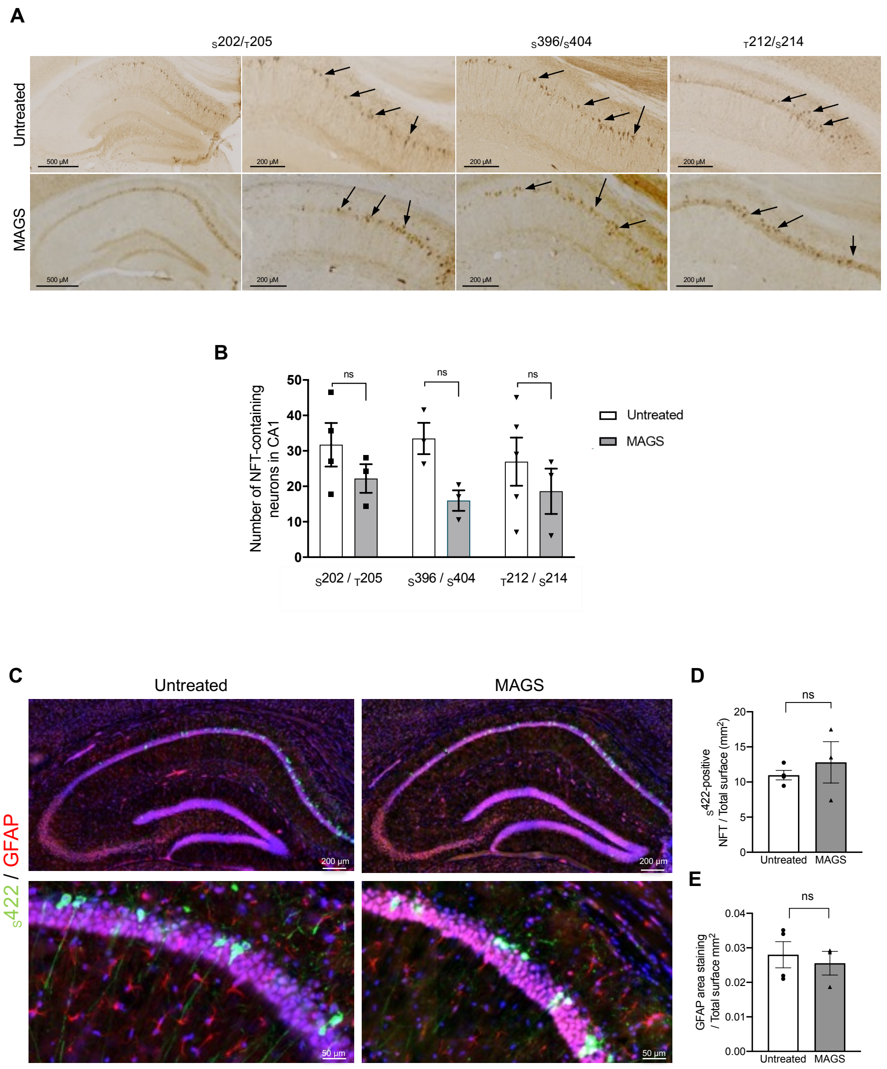

Supplementary Figure 4: Tau pathology and astrogliosis are unchanged in MAGS02-14 THY-Tau22 treated animals. A: Immunohistochemistry with the phospho-Tau antibodies against phospho-epitopes $_{S}$202/$_{T}$205 and $_{S}$396/$_{S}$404 or pathological epitopes ($_{T}$212/$_{S}$214) in the hippocampus of THY-Tau22 animals treated or not with MAGS02-14 (MAGS). **B**: The mean ± SEM number of NFT-labeled neurons observed in the CA1 of the hippocampus (3 brain slices per animal and n = 4 animals per condition) are represented on the histogram. Labeled neurons with the $_{S}$202/$_{T}$205, $_{S}$396/$_{S}$404 and $_{T}$212/$_{S}$214 were counted in the hippocampus of untreated (uncolored bars) and MAGS02-14 treated (grey bars) animals. Statistical differences were non-significant (ns). **C**: Immunofluorescence analysis of $_{S}$422-positive NFT and astrogliosis in the hippocampus of THY-Tau22 mice treated or not with MAGS02-14 (MAGS). The $_{S}$422 antibody was used for the detection of NFT (in green) and GFAP antibody was used to stain reactive astrocytes (in red). **D**: Ratio of the number of $_{S}$422-positive NFT over the total surface in mm^2 of the CA1 of the hippocampus of untreated or MAGS02-14 (MAGS) THY-Tau22 treated animals. **E**: Ratio of the glial fibrillary acidic protein (GFAP) staining over the total surface in mm^2 of the hippocampal CA1 of MAGS02-14 (MAGS) treated or untreated THY-Tau22 mice. Statistical were non-significant (ns). Results are expressed as the means ± SEM from 3 brain slices per animal and for 4 animals per condition.

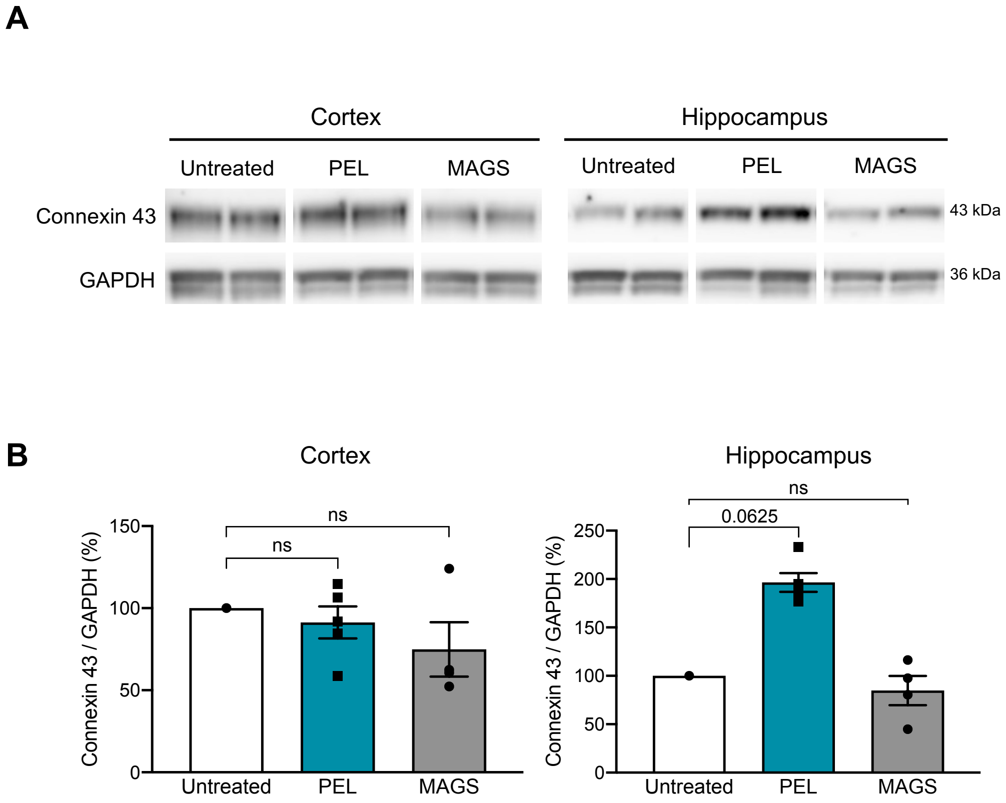

Supplementary Figure 5: Connexin 43 expression in THY-Tau22 untreated and treated animals. **A:** Western blotting of brain lysates from THY-Tau22 mice or THY-Tau22 mice treated with 1 mg/kg of PEL24-199 or MAGS02-14 was used to assess the levels of expression of Connexin43 protein. **B:** Western-blot quantification of Connexin43 expression in the hippocampus and the cortex, expressed as a percentage of the control condition. Histograms represent the means ± SEM (n = 5 mice for each condition).

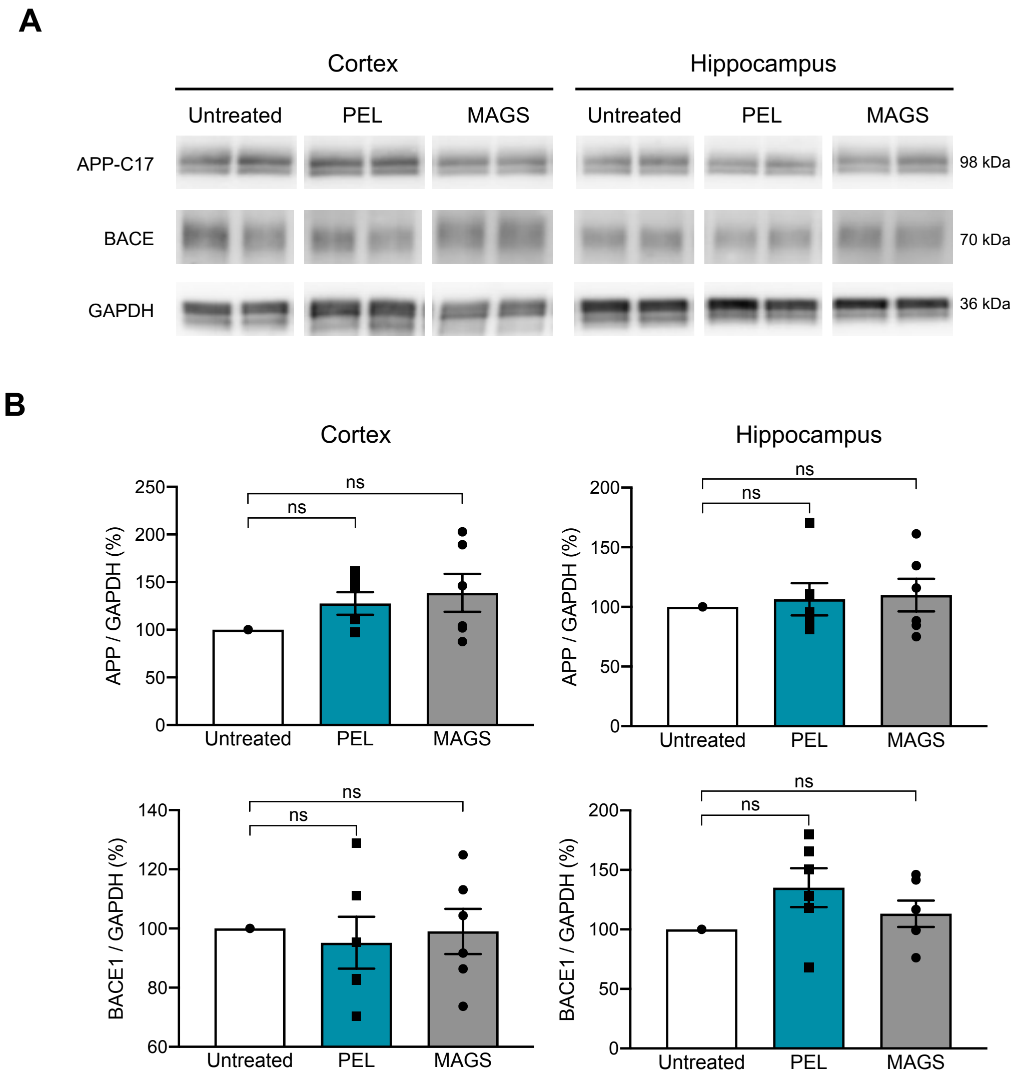

Supplementary Figure 6: PEL24-199 and MAGS02-14 did not alter the levels of expression of APP, BACE1. Brain lysates from THY-Tau22 mice (n = 5) or THY-Tau22 mice treated with 1 mg/kg of PEL24-199 (PEL, n = 5) or MAGS02-14 (MAGS, n = 5) were resolved by gels electrophoresis and blotted with APP, BACE1 (**A**). Semi-quantification of APP, BACE1 (**B**) are represented on histograms as the means ± SEM measured for samples from the cortex or hippocampus of untreated, PEL or MAGS treated animals.

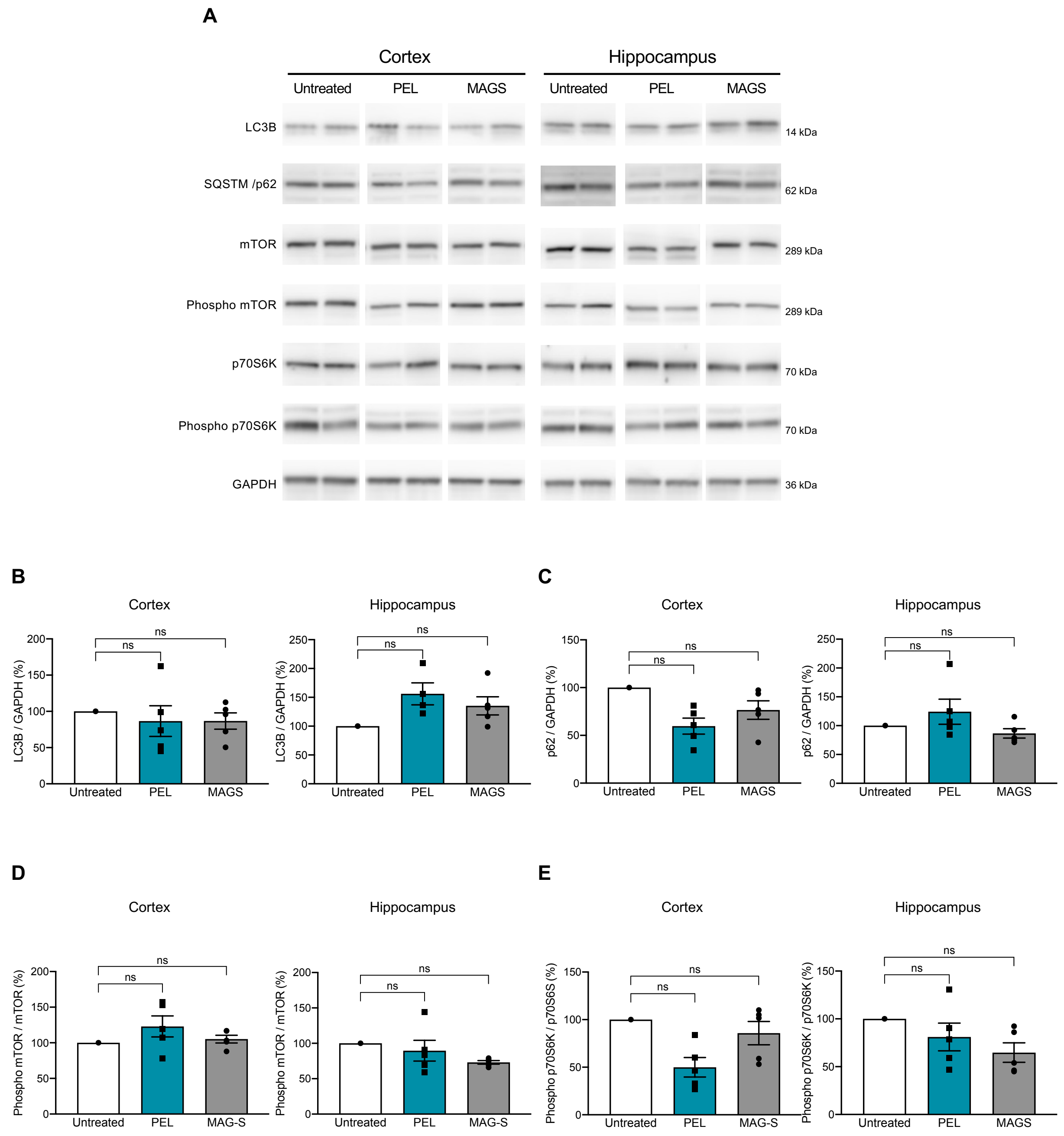

Supplementary Figure 7: PEL24-199 and MAGS02-14 did not alter the levels of expression of autophagy markers and inactivated PP2A catalytic subunit. **A**: Gel electrophoresis and blots of brain lysates from THY-Tau22 mice (n = 5) or THY-Tau22 mice treated with 1 mg/kg of PEL24-199 (n = 5) or MAGS02-14 (n = 5) were stained with antibodies against LC3B, p62, mTOR, phospho mTOR, p70S6K, phospho p70S6K. **B-E**: Histogram representations of the mean ± SEM of the semi-quantification of LC3B (**B**), p62 (**C**), phospho mTOR / mTOR ratio (**D**), phospho p70S6K / p70S6K (**E**) ration in the cortex and hippocampus of THY-Tau22 mice untreated (uncolored bars), treated with PEL24-199 (PEL) (blue bars) or MAGS02-14 (grey bars) (MAGS). A ratio phospho-mTOR/mTOR, phospho-p70S6K/p70S6K was calculated, expressed as a percentage of the control condition. Non-significant statistic tests are indicated (ns).

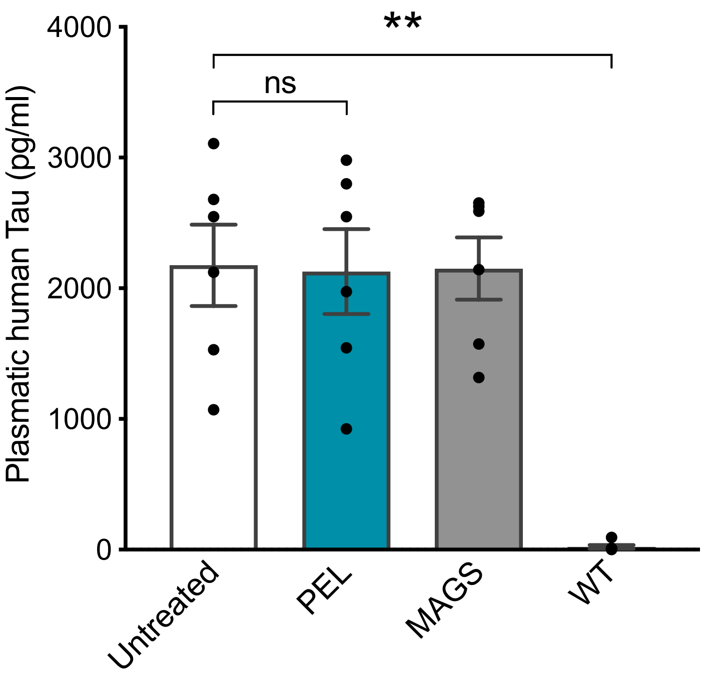

Supplementary Figure 8: Quantitative ELISA measurements of plasmatic Tau concentration of WT, untreated, MAGS02-14-treated or PEL24-199-treated THY-Tau22 mice. Histograms represent the means ± SEM (n = 7 animals per condition). Mann-Whitney statistical test was considered as significant for p values below p < 0.01 (*).
